# Influence of pain severity on the quality of life in patients with head and neck cancer before antineoplastic therapy

**DOI:** 10.1186/1471-2407-14-39

**Published:** 2014-01-24

**Authors:** Karine G Oliveira, Sandra V von Zeidler, Jose RV Podestá, Agenor Sena, Evandro D Souza, Jeferson Lenzi, Nazaré S Bissoli, Sonia A Gouvea

**Affiliations:** 1 Department of Physiological Sciences, Health Sciences Center, Federal University of Espirito Santo, Vitória, Brazil; 2 Department of Pathology, Health Sciences Center, Federal University of Espirito Santo, Vitória, Brazil; 3 Head and Neck Division - Santa Rita de Cássia Hospital – AFECC, Vitória, Brazil

## Abstract

**Background:**

The aim of this study was to assess the severity of pain and its impact on the quality of life (QoL) in untreated patients with head and neck squamous cell carcinoma (HNSCC).

**Methods:**

A study group of 127 patients with HNSCC were interviewed before antineoplastic treatment. The severity of pain was measured using the Brief Pain Inventory (BPI) questionnaire, and the QoL was assessed with the European Organization for Research and Treatment of Cancer Quality of Life Questionnaire Core-30 (EORTC QLQ-C30) and the head and neck module (QLQ-H&N35).

**Results:**

The mean age of the patients was 57.9 years, and there was a predominance of men (87.4%). The most frequent site of the primary tumor was the oral cavity (70.6%), and the majority of the patients had advanced cancers (stages III and IV). QoL in early stage of cancer obtained better scores. Conversely, the patients with advanced stage cancer scored significantly higher on the symptom scales regarding fatigue, pain, appetite loss and financial difficulties, indicating greater difficulties. Regard to the severity of pain, patients with moderate-severe pain revealed a significantly worse score than patients without pain.

**Conclusions:**

The severity of pain is statistically related to the advanced stages of cancer and directly affects the QoL. An assessment of the quality of life and symptoms before therapy can direct attention to the most important symptoms, and appropriate interventions can then be directed toward improving QoL outcomes and the response to treatment.

## Background

Head and neck cancer (HNC) comprises a group of tumors that arise in the oral cavity, pharynx and larynx. It is the 6^th^ most common cancer worldwide, accounting for 6% of cancer cases. Approximately 40% of these tumors occur in the oral cavity, 15% occur in the pharynx, and 25% occur in the larynx; in 90% of the cases, the most common histologic type is squamous cell carcinoma [1,2].

Pain is one of the several symptoms of cancer that create a poor quality of life (QoL) because pain affects physical functions and has an emotional impact [3-5]. In HNC, pain affects the oral functions and is a complaint in approximately 58% of the patients awaiting treatment and in 30% of the treated patients [4,6]. In a meta-analysis of 52 studies that calculated the prevalence of cancer pain, head and neck cancer had the highest prevalence of pain, surpassing gynecological, gastrointestinal, lung and breast tumors [7].

The complaint of pain has been reported in all clinical stages of oral cancer, with 88.1% of the cases occurring in stages III-IV. Some studies have shown a correlation between pain and tumor staging, with pain being the initial symptom in approximately 20% of the patients with oral squamous cell carcinoma [5,6].

Cancer pain is multidimensional and is directly associated with QoL [8]. The assessment of QoL has increasingly moved toward a modular approach, which allows for the evaluation of multiple dimensions of functioning. A general module, which assesses the symptoms commonly experienced by cancer patients, is supplemented by a site- or treatment-specific module that assesses difficulties unique to that particular type of cancer or treatment. Studies have confirmed that both general and site-specific measures contribute to obtaining important information concerning QoL [9].

For cancer patients, pain and symptom control are the best predictors of overall QoL scores because the effects of unrelieved pain and poorly managed symptoms have been shown to interfere with the activities of daily living, mood, mobility, and independence. Therefore, when the control of symptoms is not attended to, the QoL tends to be reduced [8,10]. Additionally, studies on the intensity of pain and QoL among patients with HNC before treatment are lacking.

We hypothesized that patients with HNC who experienced moderate to severe pain before antineoplastic treatment would report more interference with QoL scores than those patients without pain. Therefore, the purpose of this study was to assess pain severity and its impact on the QoL in untreated patients with head and neck squamous cell carcinoma (HNSCC), and assess QoL of these patients with respect to pain severity, clinical stage of the primary tumor, and lymph nodes involvement.

## Methods

### Patients

This study is prospective and controlled and it was approved by the Research Ethics Committee of the Espirito Santo Federal University (Protocol n° 99.242/2012). We interviewed 127 outpatients with primary head and neck squamous cell carcinoma consecutively who had undergone medical examinations in 2012 at the Santa Rita de Cassia Hospital-AFECC, Vitoria, ES, Brazil. The cancer patients were distributed into groups with no pain (N = 52), mild pain (N = 47), and moderate to severe pain (N = 28). Inclusion criteria were patients with untreated HNSCC aged over 18 years and both gender. The exclusion criteria were patients who had already been treated for HNSCC, had recurrent malignant disease, were unable to speak Portuguese or had a functional status sufficiently impaired to prevent answering the questionnaires. Clinical data (gender, age, tobacco and alcohol consumption, tumor location and tumor stage) were obtained from medical records.

### Assessments

The pain was measured using the item of “average pain” during the last 24 hours in the Brief Pain Inventory (BPI) [11], which was validated in the Brazilian population [12]. The pain scores were categorized into three groups according to the BPI average pain: no pain (0), mild pain (1–4), and moderate (5–6) to severe (7–10) pain [13]. The BPI asks patients to rate their pain intensity and pain interference (with general activities, mood, walking ability, normal work, relationship with others, sleep, and enjoyment of life) on an 11-point scale ranging from 0 (no pain/no interference) to 10 (as bad as you can imagine/complete interference) [11].

The QoL was assessed with the European Organization for Research and Treatment of Cancer Quality of Life Questionnaire Core-30 (EORTC QLQ-C30) version 3.0 [14], which was validated in the Brazilian population [15].

This is a 30-item questionnaire that consists of 5 functional scales (physical, role, cognitive, emotional, and social functioning), 3 symptom scales (fatigue, pain, and nausea and vomiting), a global health status/QoL scale, and a number of single items assessing additional symptoms commonly reported by patients with cancer (dyspnea, loss of appetite, insomnia, constipation, diarrhea, and financial difficulties). The patients were asked to rate each item on a 4-point scale and the global health status/QoL scale item on a 7-point scale [16].

The Quality of Life Questionnaire Head and Neck Cancer Module (EORTC QLQ-H&N35) [17] has 35 specific questions concerning problems attributed to HNC and its treatment-related side effects. There are 7 scaled answers for pain, swallowing, sensibility, speech, eating in a social setting, social contact, and sexuality. In addition,11 individual topics were evaluated taking into account the anatomic site, symptoms, and treatment (dental problems, mouth opening, dry mouth, poor salivation, coughing, sense of illness, analgesic use, nutrition difficulties, gastric tube, and weight loss or gain) [17].

All scales and single items were linearly transformed to provide a score ranging from 0 to 100; a high score on the functional scale and for global quality of life (QoL) was representative of a high level of functioning and a high QoL. However, a high score on the symptom scale represented a high level of symptomatology and problems [18]. The instruments were filled by patients with staff assistance.

### Statistical analyses

The scores from the EORTC QLQ-C30 and EORTC QLQ-H&N35 were interpreted according to the EORTC scoring manual [18]. Internal consistency in the questions was determined using Cronbach’s α coefficient, which is used as an indicator of scale reliability. The distribution of quantitative variables was determined using the mean and standard deviation (determined as normal or abnormal using the Kolmogorov-Smirnov test). An association between the domains and other factors were examined using nonparametric tests (Mann–Whitney and Kruskal-Wallis tests). Qualitative variables were analyzed using the Chi square test or Fisher’s exact test. The statistical software program SPSS version 17 for Windows (Statistical Package for the Social Sciences, Chicago, USA) was used for the data analysis. The level of statistical significance was accepted at *p* < 0.05.

## Results

The main features of our series of 127 patients with HNSCC are summarized in Table 1. The mean age of the patients was 57.9 years (range, 21–89), and there was predominance of men (87.4%). The most frequent site of the primary tumor was the oral cavity (70.6%), and the majority of the patients had advanced cancers (stages III and IV).

**Table 1:** Clinical and epidemiological features (n = 127)

**Age (years)**	
Range	21 – 89
Mean (SD)	57.9 (12.3)
	**n (%)**
**Gender**	
Female	16 (12.6)
Male	111 (87.4)
**Currently smoking**	
Yes	83 (65.4)
No	44 (34.6)
**Alcohol**	
Yes	78 (61.4)
No	49 (38.6)
**Education**	
High school or less	98 (77.2)
College or more	29 (22.8)
**Primary tumor location**	
Oral cavity	77 (60.6)
Oropharynx	28 (22.1)
Hypopharynx	4 (3.1)
Larynx	18 (14.2)
**TNM stage**	
I	25 (19.6)
II	19 (15)
III	26 (20.5)
IV	57 (44.9)
**T**	
1	25 (19.7)
2	30 (23.6)
3	24 (18.9)
4	48 (37.8)
**N**	
+	84 (66.1)
0	43 (33.9)

*Abbreviations*: *T*, Tumor size; *N*, Lymph node involvement.

The reliability coefficients (Cronbach’s α), means, and SDs for the EORTC QLQ-C30 scales are listed in Table 2. The reliability coefficient for most of the scales ranged from 0.73 to 0.89, indicating satisfactory internal consistency, while nausea/vomiting (NV) had a moderate coefficient alpha of 0.67. Only the cognitive functioning scale (CF) presented a lower coefficient (0.31). Reliability coefficients, means, and SDs for the EORTC QLQ-H&N35 are listed in Table 3. Each of the scales demonstrated a high α coefficient (>0.70), except for the speech scale (HNSP) and the social contact scale (HNSC), both of which had coefficients equal to 0.68, which is considered moderate.

**Table 2:** Descriptive analyses of the EORT QLQ–C30 items and reliability analysis

**QLQ-C30**	**Mean(SD)**	**Cronbach’s α**
Global quality of life/QoL	65.8 (27.1)	0.81
**Functional scales**		
Physical functioning	80.2 (23.3)	0.73
Role functioning	80.7 (32.3)	0.78
Emotional functioning	64.5 (33.5)	0.85
Cognitive functioning	82.1 (25.0)	0.31
Social functioning	89.8 (23.7)	0.70
**Symptom scales**		
Fatigue	21.7 (28.3)	0.75
Nausea and vomiting	5.5 (15.7)	0.67
Pain	36.1 (38.1)	0.89
Dyspnea	9.1 (23.6)	-
Insomnia	36.7 (42.7)	-
Appetite loss	31.4 (40.3)	-
Constipation	23.8 (38.9)	-
Diarrhea	2.1 (11.6)	-
Financial difficulties	30.7 (42.9)	-

**Table 3:** Descriptive analyses of the EORT QLQ–H&N35 items and reliability analysis

**QLQ–H&N35**	**Mean (SD)**	**Cronbach’s α**
Pain	30.5 (31.0)	0.78
Swallowing	32.3 (34.0)	0.86
Senses problems	15.8 (30.7)	0.85
Speech problems	23.8 (29.6)	0.68
Trouble with social eating	22.6 (28.6)	0.81
Trouble with social contact	11.6 (17.5)	0.68
Less sexuality	23.2 (36.5)	0.98
Teeth	18.1 (35.1)	-
Opening mouth	19.9 (35.2)	-
Dry mouth	22.0 (36.1)	-
Sticky saliva	41.7 (43.4)	-
Coughing	23.1 (29.2)	-
Felt ill	19.1 (34.7)	-
Pain killers	66.9 (47.2)	-
Nutritional supplements	5.5 (22.9)	-
Feeding tube	0	-
Weight loss	47.2 (50.1)	-
Weight gain	14.9 (35.8)	-

The reliability of the BPI was evaluated according to the internal consistency (Cronbach’s α coefficient). The mean score of item “average pain” during the last 24 hours in the BPI was 4.1. We separately calculated alpha coefficients for pain severity and pain interference. The internal consistency of the pain severity dimension was 0.82 and for the pain interference dimension was 0.92, indicating a satisfactory internal validity (>0.70).

The comparison of the EORTC QLQ-C30 scales with the tumor size (T) and lymph node involvement (N) indicated that the patients with an early stage tumor scored significantly higher in physical functioning (T, *p =* 0.025; N, *p =* 0.024), role functioning (T, *p =* 0.010; N, *p =* 0.004) and social functioning (T, *p =* 0,035; N, *p =* 0.002), indicating better functioning. Conversely, the patients with an advanced-stage tumor scored significantly higher on the symptom scales with regard to fatigue (T, *p =* 0.012; N, *p =* 0.003), pain (T, *p* < 0.001; N, *p =* 0.001), appetite loss (T, *p =* 0.041; N, *p =* 0.010) and financial difficulties (T, *p =* 0.039; N, *p =* 0.006), indicating greater difficulties (Table 4). On the EORTC QLQ-H&N35 scales, the patients with advanced-stage tumors had significantly higher scores on pain (T, *p* < 0.001; N, *p* < 0.001), swallowing (T, *p* < 0.001; N, *p* < 0.001), social eating (T, *p* < 0.001; N, *P* < 0.001), social contact (T, *p =* 0.005; N, *p* < 0.001), teeth (T, *p =* 0.046; N, *p =* 0.001), sticky saliva (T, *p* < 0.001; N, *p =* 0.024), pain killers (T, *p* < 0.001; N, *p =* 0.038) and weight loss (T, *p* < 0.001; N, *p* < 0.001), indicating greater impairment (Table 5).

**Table 4:** EORTC QLQ–C30 scales and TN stage

	T*					N**		
	**T1**	**T2**	**T3**	**T4**		**N0**	**N+**	
	**(n = 25)**	**(n = 30)**	**(n = 24)**	**(n = 48)**		**(n = 43)**	**(n = 84)**	
**EORTC QLQ-C30**	**Mean (SD)**	**Mean (SD)**	**Mean (SD)**	**Mean (SD)**	***p***	**Mean (SD)**	**Mean (SD)**	***p***
Emotional functioning	75.6 (27.9)	70.8 (34.1)	53.8 (35.8)	60.0 (33.0)	0.085	69.5 (31.0)	54.6 (36.2)	0.039
Physical functioning	90.4 (17.2)	84.6 (17.6)	75.2 (25.5)	74.5 (26.1)	0.025	83.8 (21.0)	73.1 (26.1)	0.024
Role functioning	97.3 (13.3)	79.4 (34.3)	77.7 (36.3)	74.3 (33.8)	0.010	86.3 (27.2)	69.7 (38.5)	0.004
Cognitive functioning	82.6 (22.8)	85.5 (18.9)	79.1 (28.7)	81.2 (27.8)	0.938	84.1 (24.1)	78.2 (26.6)	0.158
Social functioning	98.6 (6.6)	91.1 (22.6)	83.3 (23.0)	87.8 (29.1)	0.035	94.0 (19.4)	81.7 (29.0)	0.002
Global quality of life/QoL	75.6 (19.1)	69.7 (26.5)	60.7 (33.6)	60.9 (26.4)	0.130	70.3 (23.4)	57.1 (31.6)	0.033
Fatigue	9.3 (16.8)	15.1 (22.3)	25.4 (30.1)	30.5 (32.6)	0.012	16.9 (26.5)	31.2 (29.7)	0.003
Nausea and Vomiting	3.3 (10.7)	3.3 (9.1)	11.8 (24.8)	4.8 (14.9)	0.252	3.9 (13.1)	8.5 (19.7)	0.090
Pain	8.0 (16.0)	33.8 (34.3)	45.8 (44.0)	47.2 (38.4)	<0.001	27.5 (34.3)	52.7 (39.8)	0.001
Dyspnea	5.3 (12.4)	6.6 (22.1)	19.4 (33.9)	7.6 (22.0)	0.124	9.1 (23.9)	9.3 (23.3)	0.816
Insomnia	25.3 (36.3)	32.2 (39.6)	30.5 (43.8)	48.6 (45.5)	0.129	30.5 (39.4)	48.8 (46.7)	0.041
Appetite loss	17.3 (33.4)	24.4 (40.9)	38.8 (41.3)	39.5 (41.0)	0.041	25.3 (38.5)	43.4 (41.4)	0.010
Constipation	9.3 (29.0)	25.5 (38,8)	33.3 (43.9)	25.6 (40.2)	0.127	20.2 (36.2)	31.0 (43.2)	0.135
Diarrhea	0	1.1 (6.0)	5.5 (21.2)	2.0 (10.6)	0.511	0.7 (5.1)	4.6 (18.6)	0.199
Financial difficulties	12.0 (27.0)	24.4 (40.9)	45.8 (48.9)	36.8 (44.6)	0.039	22.6 (38.0)	46.5 (47.7)	0.006

*Kruskal-Wallis test, **Mann Whitney test. *Abbreviations*: *T*, Tumor size; *N*, Lymph node involvement.

**Table 5:** EORTC QLQ-H&N35 scales and TN stage

	T*					N**		
	**T1**	**T2**	**T3**	**T4**		**N0**	**N+**	
	**(n = 25)**	**(n = 30)**	**(n = 24)**	**(n = 48)**		**(n = 43)**	**(n = 84)**	
**EORTC QLQ –H&N35**	**Mean (SD)**	**Mean (SD)**	**Mean (SD)**	**Mean (SD)**	***p***	**Mean (SD)**	**Mean (SD)**	***p***
Pain	7.6 (13.5)	29.4 (26.5)	28.4 (28.2)	44.2 (34.5)	<0.001	24.0 (29.2)	43.4 (30.7)	<0.001
Swallowing	2.0 (6.0)	32.7 (32.8)	31.2 (33.5)	48.4 (33.3)	<0.001	22.8 (32.0)	50.9 (30.1)	<0.001
Senses problems	15.3 (33.6)	10.0 (21.2)	16.6 (28.2)	19 (35.4)	0.926	14.6 (30,6)	18.2 (31.2)	0.362
Speech problems	13.7 (25.7)	15.1 (22.5)	30.5 (33.4)	31.2 (31.2)	0.029	20.8 (27.9)	29.7 (32.2)	0.240
Trouble with social eating	2.0 (6.9)	19.1 (26.6)	25.6 (29.8)	34.0 (30.5)	<0.001	14.4 (23.5)	38.5 (31.2)	<0.001
Trouble with social contact	5.0 (7.2)	7.7 (14.0)	18.8 (20.4)	13.8 (20.1)	0.005	7.4 (10.7)	19.8 (24.2)	0.001
Less sexuality	12.0 (25.2)	16.1 (29.1)	29.1 (43.1)	30.5 (40.5)	0.211	14.0 (29.1)	41.0 (42.9)	<0.001
Teeth	4.0 (14.6)	16.6 (34.7)	12.5 (29.1)	29.1 (42.1)	0.046	10.3 (26.8)	33.3 (43.6)	0.001
Opening mouth	1.3 (6.6)	10.0 (27.8)	19.4 (30.9)	36.1 (42.8)	<0.001	15.4 (30.3)	28.6 (42.1)	0.112
Dry mouth	25.3 (38.8)	27.7 (37.2)	18.0 (36.7)	18.7 (34.3)	0.416	22.2 (36.7)	21.7 (35.5)	0.946
Sticky saliva	13.3 (27.2)	37.7 (40.8)	34.7 (45.5)	62.5 (41.6)	<0.001	35.3 (40.5)	54.2 (46.5)	0.024
Coughing	20.0 (25.4)	26.6 (33.2)	22.2 (27.2)	22.9 (30.0)	0.955	21.4 (28.6)	26.3 (30.4)	0.337
Felt ill	8.0 (19.9)	11.1 (26.7)	23.6 (38.6)	27.7 (40.8)	0.125	13.8 (29.3)	29.4 (41.9)	0.042
Pain killers	28.0 (45.8)	70.0 (46.6)	83.3 (38.0)	77.0 (42.4)	<0.001	60.7 (49.1)	79.0 (41.1)	0.038
Nutritional supplements	8.0 (27.6)	6.6 (25.3)	0	6.2 (24.4)	0.613	4.7 (21.4)	6.9 (25.7)	0.606
Feeding tube	0	0	0	0	-	0	0	-
Weight loss	4.0 (20.0)	40.0 (49.8)	54.1 (50.8)	70.8 (45.9)	<0.001	34.5 (47.8)	72.0 (45.3)	<0.001
Weight gain	28.0 (45.8)	23.3 (43.0)	8.3 (28.2)	6.2 (24.4)	0.035	16.6 (37.4)	11.6 (32.4)	0.453

*Kruskal-Wallis test, **Mann Whitney test. *Abbreviations*: *T*, Tumor size; *N*, Lymph node involvement.

**Figure 1:**
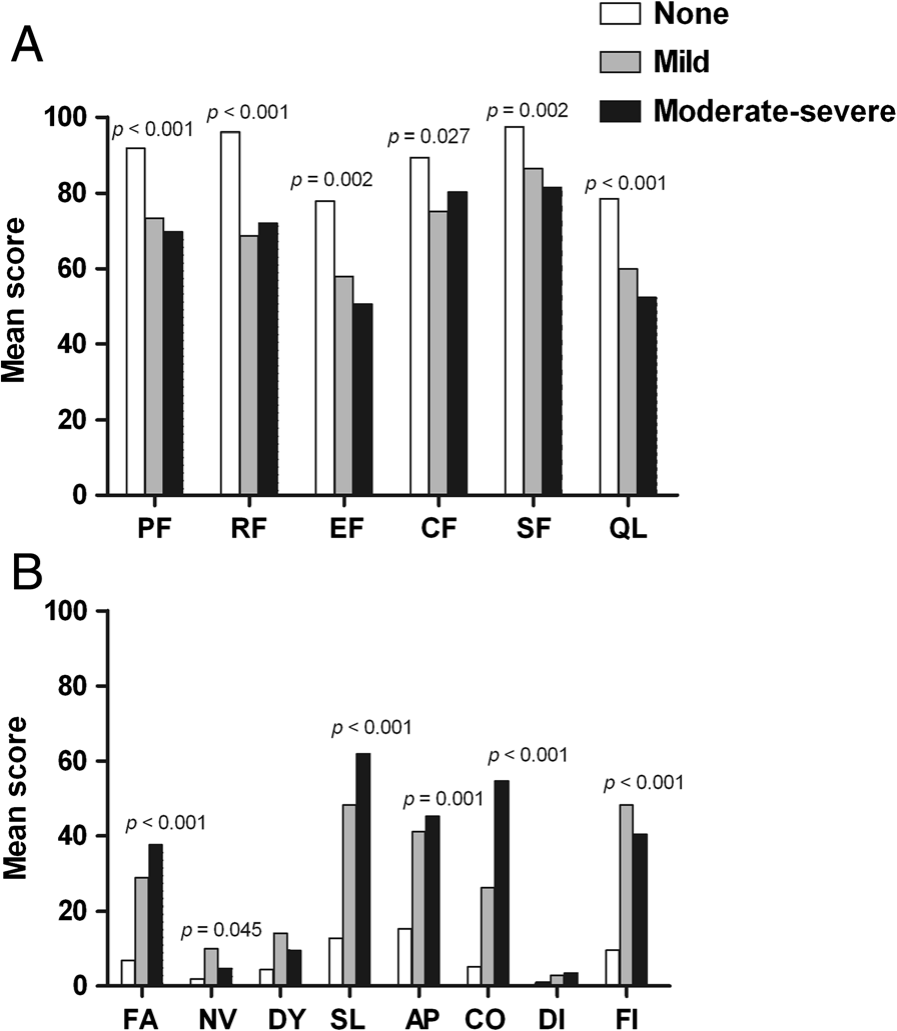
**Mean scores on the EORTC QLQ-C30 for patients with head and neck cancer according to the level of pain during the last 24 h (BPI-Average pain intensity).** The scales are as follows: PF, physical functioning; RF, role functioning; EF, emotional functioning; CF, cognitive functioning; SF, social functioning; QL, global quality of life; FA, fatigue; NV, nausea/vomiting; DY, dyspnea; SL, insomnia; AP, appetite loss; CO, constipation; DI, diarrhea; and FI, financial difficulties. For Figure 1**A**, higher scores reflect better functioning; for Figure 1**B**, higher scores indicate worse functioning. Significance was determined by Kruskal-Wallis tests.

Significant differences in the EORTC scales were found with regard to pain intensity. On the EORTC QLQ-C30, the cancer group without pain had better scores on all of the functional scales: physical functioning (PF, *p* < 0.001), role functioning (RF, *p* < 0.001), emotional functioning (EF, *p =* 0.002), cognitive functioning (CF, *p =* 0.027), social functioning (SF, *p =* 0.002) and global quality of life (QL, *p* < 0.001) (Figure 1A). However, with regard to the symptom scales, the cancer group with moderate-severe pain indicated greater impairment on the fatigue (FA, *p* < 0.001), insomnia (SL, *p* < 0.001), appetite loss (AP, *p =* 0.001) and constipation (CO, *p* < 0.001) scales (Figure 1B). The cancer group with mild pain showed greater impairment on the nausea/vomiting (NV, *p =* 0.045) and financial difficulties (FI, *p* < 0.001) scales when compared with the cancer group with no pain.

**Figure 2:**
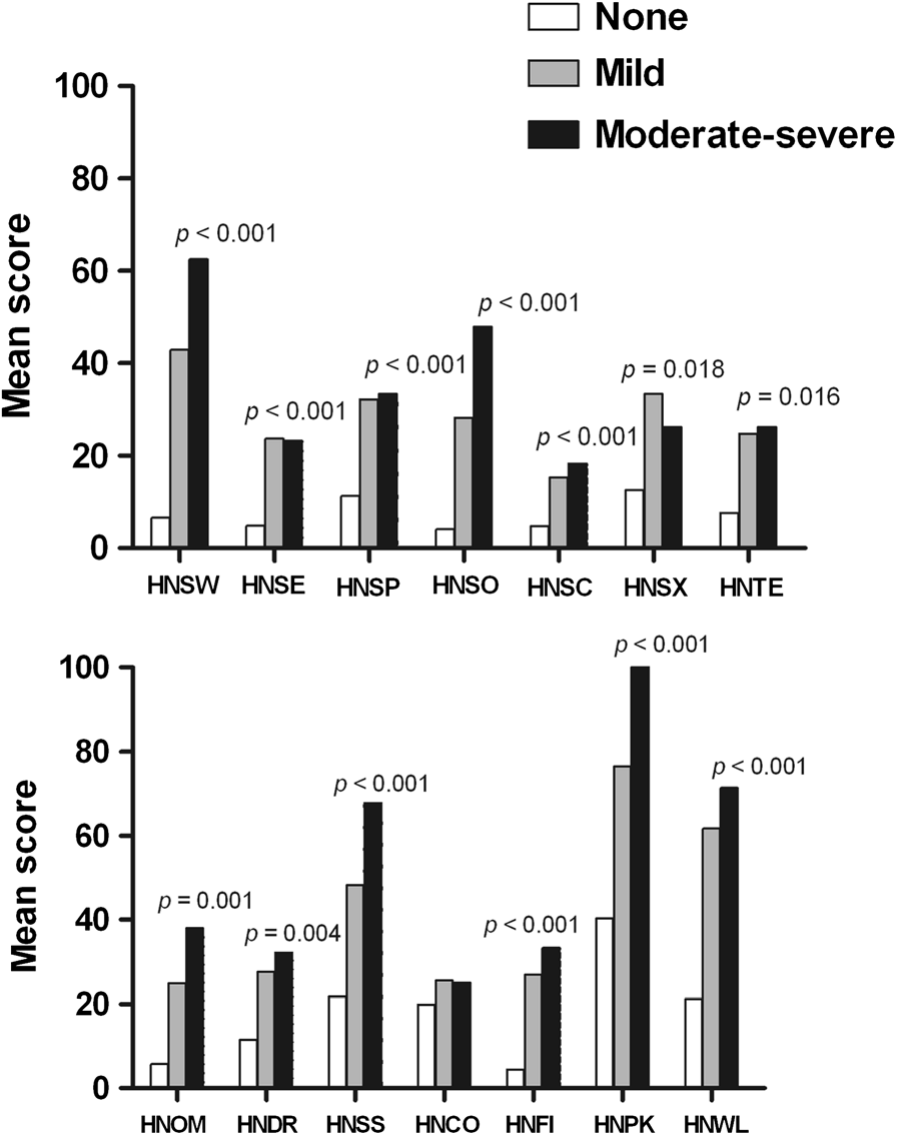
**Mean scores on the EORTC QLQ-H&N35 for patients with head and neck cancer according to the level of pain during the last 24 h (BPI-average pain intensity).** The scales are as follows: HNSW, swallowing; HNSE, senses; HNSP, speech; HNSO, social eating; HNSC, social contact; HNSX, sexuality; HNTE, teeth; HNOM, opening mouth; HNDR, dry mouth; HNSS, sticky saliva, HNCO, coughing; HNFI, felt ill; HNPK, pain killers; and HNWL, weight loss. Higher scores indicate poorer functioning. Significance was determined by Kruskal-Wallis tests.

The EORTC QLQ-H&N35 questionnaire also demonstrated significantly worse scores on the swallowing (HNSW, *p* < 0.001), speech problems (HNSP, *p* < 0.001), social eating (HNSO, *p* < 0.001), social contact (HNSC, *p* < 0.001), teeth (HNTE, *p =* 0.016), opening mouth (HNOM, *p =* 0.001), dry mouth (HNDR, *p =* 0.004), sticky saliva (HNSS, *p* < 0.001), feeling ill (HNFI, *p* < 0.001), pain killers (HNPK, *p* < 0.001) and weight loss (HNWL, *p* < 0.001) scales for the cancer group with moderate-severe pain (Figure 2). In each case, the HNSCC patients with moderate-severe pain reported greater difficulties than those with mild or no pain. The group with mild pain presented scores that were significantly worse than the no pain group on the senses problems (HNSE, *p* < 0.001) and less sexuality (HNSX, *p =* 0.018) scales.

**Figure 3:**
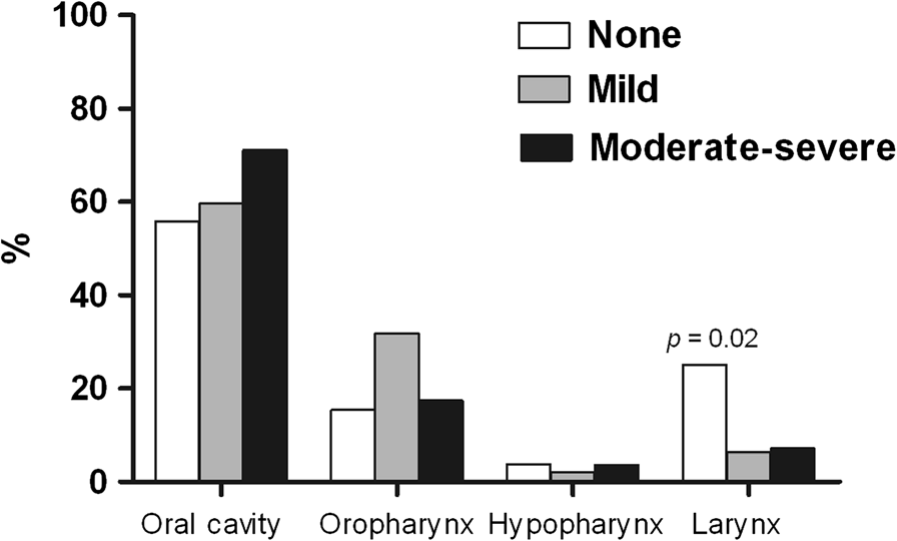
Correlation between pain intensity (BPI-average pain intensity) and anatomic sites.

**Figure 4:**
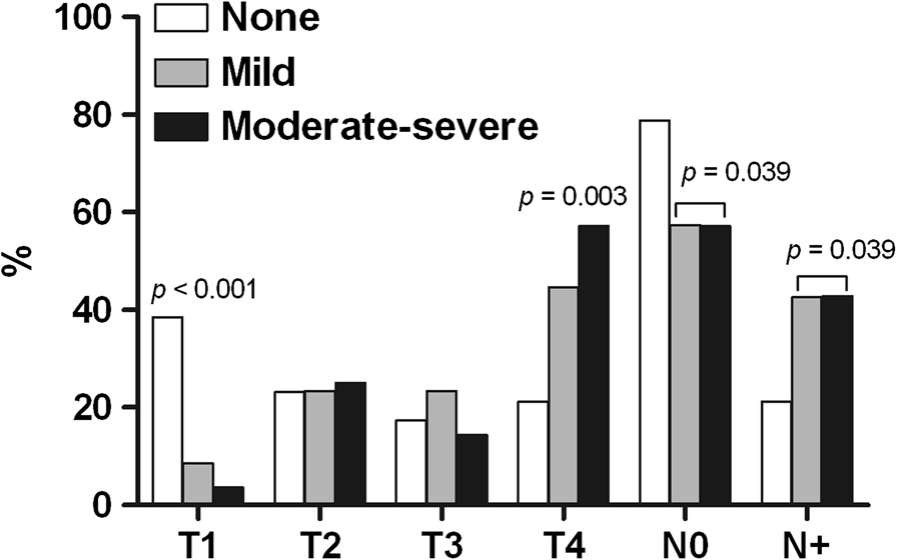
**Correlation between pain intensity (BPI-average pain intensity) and TN stage.** T: tumor size; N: lymph node involvement.

The intensity of pain was not correlated with the tumor location (Figure 3); however, the group with larynx tumors had more patients without pain (*p =* 0.02). Analyzing the tumor classification, the group with T1 tumors had more patients with no pain (*p* < 0.001), conversely, patients with T4 tumors indicated a greater intensity of pain (*p =* 0.003). The absence of lymph node involvement (N0) revealed a difference in the percentage of patients without pain (*p* = 0.039) (Figure 4).

## Discussion

This is the first study to evaluate the pain severity among untreated HNSCC patients and its impact on QoL. We hypothesized that the intensity of pain in patients with untreated HNSCC may be significantly correlated with a poor QoL. Patients with an advanced-stage tumor showed higher impairment in functional status (physical, role and social functioning) and worse symptoms, which is in accordance with the results of earlier studies [19-21] and demonstrates the strong correlation between tumor stage and QoL. Patients with an early-stage tumor had less pain compared with those who had an advanced-stage tumor. Regarding tumor site, although more patients with a tumor in the oral cavity indicated that they had moderate to severe pain, this difference was not significant and is difficult to compare with prior results because of the lack of earlier studies regarding the impact of pain intensity in pretreatment HNSCC patients.

The HNSCC patients with moderate to severe pain reported higher levels of interference on all of the functioning scales, the global QoL and the 4 symptoms (fatigue, pain, appetite loss and financial difficulties) on the EORTC QLQ-C30 scales, whereas patients without pain indicated better results on those scales. Thus, increasing pain is related to a reduced quality of life and increased symptoms. We found similar results with the EORTC QLQ-H&N35 because the 11-symptom scales revealed the worst scores (indicating a high level of problems) for patients with moderate to severe pain.

In our study, although 66.9% of all patients reported that they had used analgesic medication for pain control, the number of patients with pain (59%) remained high, similar to what was found in an earlier study [22]. While we did not evaluate the analgesic efficacy or regimens, the persistence of pain may reflect the possibility that it is difficult for patients to report their symptoms to a physician or may suggest that the patients’ medication may not be adequately effective. Patients with head and neck, gastrointestinal and thoracic malignancies are more likely to experience severe pain compared with patients with other tumors (52.6%, 33.9% and 30.5%, respectively) [23]. Another study has shown that 58% of HNC patients felt that it was necessary to fill in the QoL questionnaire before their visit because this would help them to describe their symptoms to their doctors [24].

Normally, cancer pain is classified into three categories: pain caused by tumor growth, pain caused by treatment, and pain unrelated to cancer [25]. Therefore, we excluded pain caused by treatment because the evaluation of our patients was performed before of any type of cancer treatment. Tumor growth may cause pain by compressing and invading surrounding tissues, including muscles, bones, and peripheral nerves. Additionally, the head and neck have a rich blood supply and a large numbers of nerves that may affect tumor growth and pain [25,26].

A potential limitation of our study might be that we chose to use the BPI average pain intensity item as the pain intensity criterion, the study was performed with moderate sample, and patients’ recruitment was from one center. However, evaluating the level of pain experienced most frequently is more important for assessing pain’s interference than evaluating shorter periods with the highest/lowest pain intensities. Additionally, this criterion is in accordance with other studies [27-30], and the BPI is recommended by the European Association of Palliative Care as a pain assessment tool in clinical studies [31]. As in prior studies, each of the questionnaire scales demonstrated acceptable reliability, with the exception of the cognitive functioning scale, which has been problematic [9,14,32-35].

Although head and neck cancer has the highest prevalence of pain [7], clinical health care professionals focus on the preparation for surgery and issues of the immediate post-operative period, and the management of symptoms is neglected [36]. Furthermore, palliative care is initiated only for end-stage cancer patients, and the mean time from the initiation of palliative care to death is 21.9 days in head and neck cancer patients, suggesting that incurable patients may be referred to palliative care institutions too late. The majority of patients (85%) admitted to palliative care had inadequate pain control prior to admission [37]. Therefore, an evaluation of patients before the initiation of anti-cancer therapy is important because most studies have focused on the analysis of pain during or after treatment.

## Conclusions

Assessing the quality of life and symptoms before therapy can direct attention to the most important symptom, such as pain, and thus, appropriate interventions can improve QoL outcomes and the response to treatment.

## Abbreviations

QoL: Quality of life; HNC: Head and neck cancer; HNSCC: Head and neck squamous cell carcinoma; EORTC QLQ-C30: European Organization for Research and Treatment of Cancer Quality of Life Questionnaire Core-30; QLQ-H&N35: Quality of Life Questionnaire Head and Neck Cancer Module; BPI: Brief Pain Inventory; SPSS: Statistical Package for the Social Sciences; T: Tumor size; N: Lymph node involvement; PF: Physical functioning; RF: Role functioning; EF: Emotional functioning; CF: Cognitive functioning; SF: Social functioning; QL: Global quality of life; FA: Fatigue; NV: Nausea/vomiting; DY: Dyspnea; SL: Insomnia; AP: Appetite loss; CO: Constipation; DI: Diarrhea; FI: Financial difficulties; HNSW: Swallowing; HNSE: Senses; HNSP: Speech; HNSO: Social eating; HNSC: Social contact; HNSX: Sexuality; HNTE: Teeth; HNOM: Opening mouth; HNDR: Dry mouth; HNSS: Sticky saliva; HNCO: Coughing; HNFI: Felt ill; HNPK: Pain killers; HNWL: Weight loss.

## Competing interests

The authors declare that they have no competing interests.

## Authors' contributions

KGO, SVVZ, NSB, SAG: contributed to the conception and design of the study, analysis of data and critically read the manuscript. JRVP, AS, EDS, JL: collected data and critical revision. All contributing authors have no disclosures to make. All authors read and approved the final manuscript.

## Pre-publication history

The pre-publication history for this paper can be accessed here:

http://www.biomedcentral.com/1471-2407/14/39/prepub
